# Employee Management and Animal Care: A Comparative Ethnography of Two Large-Scale Dairy Farms in China

**DOI:** 10.3390/ani11051260

**Published:** 2021-04-27

**Authors:** Maria Chen, Marina A. G. von Keyserlingk, Sabina Magliocco, Daniel M. Weary

**Affiliations:** 1 Animal Welfare Program, Faculty of Land and Food Systems, University of British Columbia, 2357 Main Mall, Vancouver, BC V6T 1Z4, Canada; mchen242@mail.ubc.ca (M.C.); nina@mail.ubc.ca (M.A.G.v.K.); 2 Department of Anthropology, University of British Columbia, Vancouver, BC V6T 1Z1, Canada; smaglioc@mail.ubc.ca

**Keywords:** animal welfare, farm culture, human resource, behavior change, qualitative methods

## Abstract

**Simple Summary:**

China’s dairy industry is growing and restructuring to favor large-scale dairy farms. The lead author lived on two large-scale dairy farms in China and conducted immersive fieldwork where she participated in daily cattle care alongside the workers, conducted interviews with workers, and collected relevant documents. We found that employee management critically shaped animal care on both farms. Workers reported improvements to animal care on both farms, attributing these to improved employee management practices. Our findings suggest that cattle care may be improved through employee management practices such as fostering a positive organization culture, ensuring better working conditions, and incentivizing farm workers.

**Abstract:**

Farm management can directly and indirectly affect animal care. We explored how farm management affected animal care on two large dairy farms in China (anonymized as Farm A and Farm B). We used a mini-ethnographic case study design whereby the first author lived for 38 days on Farm A and 23 days on Farm B. She conducted participant observation and ethnographic interviews with farm staff positions within five departments in Farm A and six departments in Farm B. In addition, she conducted 13 semi-structured interviews (seven on Farm A; six on Farm B). We used template analysis to generate key themes. On both farms, workers believed that animal care practices had improved over time, due to three key employee management factors: 1) organizational culture, 2) competency of worker and management, and 3) an effective incentive system. Our results suggest that animal care may be improved in this context by: 1) promoting a culture in which workers have ‘grit’ and are eager to learn, 2) ensuring basic worker wellbeing, and 3) using animal care outcomes as performance indicators linked to pay.

## 1. Introduction

The changes in technology, genetics, housing, and management associated with increased farm size have resulted in benefits and challenges to dairy cattle welfare [1]. Commonly discussed welfare issues affecting dairy cattle include those associated with health (e.g., lameness, mastitis), negative affective states such as pain (e.g., due to procedures like dehorning), and the inability to express key natural behaviours (e.g., grazing and maternal-young interactions) [2]. While poor animal health can lead to reduced production, production alone is not a reliable indicator of welfare, in part because higher production can be associated with a higher incidence of health disorders [3].

Fraser [4] points out that the study of animal welfare and what it means to provide “a good life for animals” is shaped by cultural context and values. Attitudes towards animals in China are complex and nuanced. While Confucianism is anthropocentric [5], it promotes the virtue of ren (仁, compassion and kindness) towards non-human animals. Taoism emphasizes harmonious existence of man within nature [6]. Buddhists promote respect for animals [7]. Cao [8] argues that the concept of ‘animal welfare’ is compatible with major traditional Chinese philosophies. Although such ideals are not always expressed in modern society [8], public reaction to highly publicized animal cruelty cases indicates that there is growing concern for treatment of animals [9].

The term ‘animal welfare’ was introduced to China in the 1990s as 动物福利 (动物 meaning animal; 福利 meaning welfare) [10]. China’s focus on this area thus lags around 30 years behind Western counties [11]. Shi [12] suggests that the direct translation of ‘animal welfare’ and its simplistic explanations during initial promotion led to misunderstanding. Presently, there is low but increasing interest in animal welfare by the Chinese government [13,14], agricultural industry leaders [15], students [16,17], and the general public [18,19], and an increasing body of Chinese academic literature that explores questions related to animal welfare [12,20].

To improve farm animal welfare, it is critical to understand and engage livestock stakeholders whose decisions impact their animals [21]. Work to date has used surveys and focus groups to understand Chinese stakeholder attitudes [22,23,24], perceptions [15,25], and knowledge [26,27]. However, Burton et al. [28] argued that attitudes and knowledge of individuals play a minor role compared to what they termed “cowshed culture”, i.e., understanding the culture and context on farm provides insights into what shapes actual behaviors.

Human resource management (e.g., affecting the skills, knowledge, and motivation of workers) is critical in addressing farm animal welfare issues [29]. Although there is research about human resource management in the dairy industry [30], no work to date has focused on how employee management impacts animal care practices in a Chinese context. Ethnography (a set of methods for understanding cultural and social worlds [31]) provides insight into participants’ experiences and the farm culture.

We used mini-ethnographic case studies to explore factors influencing animal care on two large dairy farms in China, with a special focus on factors related to employee management. The phrase ‘animal care’ was used during this study, avoiding the use of the term ‘animal welfare’ that would likely have been less familiar to participants.

## 2. Materials and Methods

### 2.1. Mini-Ethnographic Case Study

As ethnographies have rarely been used in animal welfare research [32], we first describe ethnography and clarify what our study intends to achieve. Ethnographies are methods used to understand social groups and involve immersive fieldwork and participant observation [31]. MC actively engaged and participated in daily farm life to understand insider perspectives of farm workers. Strengths and limitations of this approach are explored in Section 4.4. Mini-ethnographic case studies typically last a few weeks and focus on a few cases [33].

Ethnographic research utilizes an inductive and exploratory approach: our initial goal was to explore factors influencing animal care, and through fieldwork we found that employee management shaped animal care on both farms. Thus, our study focused on how employee management shaped animal care on the two farms MC visited.

Though our study focuses on animal care, our participants only included humans; observations of animals occurred only tangentially when participants interacted with them (e.g., during herding, milking, feeding), thus we do not make direct claims about animal welfare on these farms. Inferences about animal care practices are the result of MC’s observations, available farm data, and conversations with workers.

China is a geographically and culturally diverse country, and ethnographic data are based on locally relevant anecdotes and observations. As such, findings are not intended to be generalizable. However, we can draw useful conclusions using thorough analysis (see Section 2.5) and strategies to increase trustworthiness (see Section 2.6). We make claims specific to farms we studied, but with an understanding of these practices and context, our conclusions may be transferred to similar contexts.

### 2.2. Research Ethics

This study was approved by The University of British Columbia’s Behavioral Research Ethics Board under #H18-03664. Farms and participants are anonymized to protect their identities. Legal representatives of both farms provided written consent and farm access, while participants provided verbal consent.

### 2.3. Farm Context

Ethnographic findings are specific to the culture under study, so we first provide relevant context necessary for understanding data generation and results. Farm A and Farm B were selected based on meeting the inclusion criteria of being large Chinese dairy farms (herd size > 500 cattle; [34]), feasibility of access [31], and ability to allow for cross-case comparison [35]. MC’s mutual connections introduced her to the legal representatives of both farms who then granted access.

Farm A (herd size ~1500; ~50 farm employees) is located in central China. It is the only farm owned by Company A, a private dairy processing company who sources most of its milk from small farms nearby. Farm B (herd size ~11,000; ~200 farm employees) is located in east China. At the time of writing, it was the newest and largest farm owned by state-owned dairy company. Overall, Farm B workers had more qualifications and were generally younger compared to Farm A (see Table 1).

### 2.4. Data Generation

MC paid an introductory 5 day visit to Farm A in April 2019, and later conducted fieldwork for 38 days on Farm A (27 September to 4 November 2019), and 23 days on Farm B (11 November to 3 December 2019).

MC conducted daily participant observation, ethnographic interviews (unstructured conversations), and critical reflections which all generated fieldnotes. Participant observation involved participating in the daily activities of the workers to gain insight into common animal care practices on the farms. Purposive sampling was used to identify participants who could discuss factors influencing animal care.

On Farm A, MC conducted participant observation with 20 employees including the farm management team (farm manager, deputy farm manager, 2 veterinarians, reproductive specialist, and nutritionist), and members from the 5 farm teams (maternity, calf and heifer, milking parlor, reproductive, waste management).

On Farm B, MC conducted participant observation with 21 workers from the farm management team, various department leaders, team leaders, and farm workers. Participant observation took place across 6 out of 8 departments: data collection, veterinary, maternity, calf and heifer, milking parlor, and reproductive. Due to limited time on this farm, MC did not conduct participant observation with nutrition and environmental department (waste management and recycling of manure).

Participant observation with each department lasted between 2 and 8 days, dependent on the participants’ comfort level and their contribution to data generation. Participation ranged from observer (witnessing procedures such as surgery and hoof trimming) to complete participant (taking part in calf feeding, milking, manure cleaning). MC participated as much as possible to try gaining an insider perspective, given her own comfort level, skill level, and relationship with the worker.

Ethnographic interviews were conducted in Mandarin by MC and the participant’s preferred Chinese dialect. These dynamic, purposeful conversations were open-ended, but often focused on the research topic and the daily activities [31]. For example, during calf feeding, MC asked, “why are we feeding this amount?” and “how do you know this is the right amount?” to understand worker decision-making.

Throughout fieldwork, MC took descriptive, handwritten fieldnotes on conversations, setting, and verbal and non-verbal interactions between cattle and humans. Descriptive fieldnotes were a mixture of English and Chinese to capture local phrases and meaning.

Since MC was integral to the data generation process, she wrote daily reflective fieldnotes to critically reflect on how her identity shaped her findings. For example, her identity as a female, Chinese-Canadian graduate student studying in an animal welfare program with little experience working on a dairy farm shaped her relationships and interactions with workers.

In addition, MC conducted 13 semi-structured, audio-recorded interviews (7 on Farm A; 6 on Farm B) using an established interview guide (see Table S1 in supplementary materials). These interview questions focused on worker background, perception of cattle well-being, and animal care on the farm. MC also collected relevant documents (e.g., standard operating procedures, SOPs, which were made available on Farm B).

### 2.5. Data Analysis

MC transcribed all interviews, then uploaded and organized fieldnotes, documents, and interview transcripts in NVivo 12.6.0 (QSR International, Vancouver, BC, Canada).

MC used template analysis to analyze all data [36]. As a type of thematic analysis [37], the goal was to examine all data and eventually develop ‘themes’, or overarching patterns and conclusions relevant to the research topic.

Following King and Brooks [36], MC first familiarized herself with the data by transcribing the interviews, and re-reading documents and fieldnotes. MC then selected 5 days of data and interviews which involved diverse participant roles and contained rich data relevant to the research topic of employee management’s influence on animal care. Next, MC started preliminary ‘coding’ of this subset of data. ‘Coding’ was a process of labelling segments of text with a relevant phrase called a ‘code’ (e.g., “eager to learn”). This list of codes was compiled into a ‘template’, where related codes were ‘clustered’ together and arranged in a hierarchical way. For example, the codes “eager to learn” and “responsibility” fell under the ‘parent code’ of “work ethics”, under the larger theme of “competent workers”.

The rest of the data were then coded using this initial template as a starting point. MC closely read the remaining data, refining, adjusting, and adding to the codes in the template based on what she interpreted from the data. Once no more major codes were added to the template, the final template was used to complete a final coding of the whole dataset (see Table S2 in supplementary materials for final template).

To help generate findings, MC also created a document to note down data that were similar, different, contradictory, or surprising to her. Through template analysis, MC generated three major themes relevant to how employee management shaped animal care.

### 2.6. Trustworthiness

MC used several strategies to improve trustworthiness of findings in accordance with Nowell et al. [38]. To improve credibility of the data, MC engaged in prolonged participation in, and observation of participants’ daily life. Data was triangulated from a variety of sources (observations, MC’s experiences, interviews with multiple individuals). During analysis, MC created an audit trail by keeping successive versions of the templates accompanied by commentary to changes (see supplementary material B for sample). MC continued to write reflexive journal entries throughout analysis (entries written after fieldwork were not analyzed). To reduce meaning being lost in translation, quotes were translated during final write-up. MC’s translations were checked by four individuals fluent in Mandarin and English.

### 2.7. Data Representation

Findings are summarized into three themes, each reported using relevant fieldnote and transcript excerpts. Longer excerpts are indented and where translations could be ambiguous, we provide the original Chinese in brackets. Original Chinese text to longer quotes and excerpts are presented in supplementary materials A. Participants are identified in the format ‘A1′, with the letter representing their farm, and the number as an anonymous identifier of individuals within that farm. The participant role and identifier are occasionally omitted to protect the anonymity of participants.

## 3. Results

We begin by describing contextual factors related to animal care on Farm A and B and then present the 3 themes of how employee management improved animal care: 1) organizational culture, 2) competent workers and management, and 3) an effective incentive system. For the first theme the organizational culture is described separately for each farm, but for the second and third themes the findings on both farms are described together to ease comparison.

### 3.1. Animal Care Context

Farm A’s workers reported that the CEO hired a new farm management team in March 2019 in response to a series of challenges. Workers reported that during the previous winter (i.e., before the change in management), on average one cow and one calf died each day (A18, cleaner; A7, calf caretaker). Other than high mortality, a farm management team member (A12) mentioned “there was low productivity, many ill and emaciated cattle, mastitis and metritis were left untreated”. Lameness was high, estimated by a farm management team member (A13) to be more than 50%. By the time of MC’s visit in September 2019, workers commented that the new farm management had brough about improvements in animal care, including reduced mortality and morbidity (A5, calf veterinarian; A7, calf caretaker), mastitis and metabolic diseases (A11, maternity veterinarian), improved productivity and hoof health (A14, milker), a cleaner barn environment, and improved heifer growth (A19, heifer barn cleaner).

Relatively speaking, Farm A’s average daily yield was 25 kg/cow/day in November 2019, slightly lower than the 2018 averages for China (26.2 kg/cow/day) and Chinese dairy farms with ≥1000 cattle (30.9 kg/cow/day) [39]. High somatic cell count (SCC) is often an indicator of mammary gland infections [40], and management reported SSC to be over 400,000/mL, higher than the 2018 averages for China (262,000/mL) and Chinese dairy farms with ≤1000 cattle (237,000/mL).

Farm B’s lactating cattle produced on average 34 kg/cow/day in November 2019, higher than the 2018 averages for China and Chinese dairy farms with ≤1000 cattle. SSC in November 2019 was 130,000/mL, lower than the 2018 averages for China and Chinese dairies with ≤1000 cattle. Unlike Farm A, Farm B kept records that enabled them to report that in 2019 they had a 99% survival rate for calving and a clinical lameness prevalence of 2%. Clinical mastitis prevalence was reported below 2%, much below the national average estimated in 2019 (33.4%) [41].

Farm B’s workers did not report major shifts in farm management prior to November 2019. According to workers, productivity (B2, data management department member), calf growth and health (B14, calf caretaker), fertility (B1, member of farm management; B7, department leader), and barn cleanliness (B15, department leader) have steadily improved since the farm started operating.

### 3.2. Organizational Culture

Schein [42] defined organizational culture as the visible artifacts, beliefs, values, and assumptions of an organization. We found organizational culture, shaped by the leader of a group (company level, farm level, and department level), seemed to affect animal care. This section explores aspects of organizational culture which were deemed to substantially contribute to animal care outcomes. Farm B’s section is longer as we describe how the Chinese Communist Party (CCP) culture influenced the state-owned farm culture and provide an example where the unique reproductive departmental subculture influenced animal care.

#### 3.2.1. Organizational Culture: Farm A

According to a member of the new farm management team (A12), “organizational culture is the soul of an organization, something that unites people’s hearts to do things”. Company culture seem determined by company-level leadership (some are the CEO’s relatives), which the farm management team (without familiar ties to the CEO) perceived were not under their influence. The poor company culture was perceived to negatively impact animal care and limit the farm management’s ability to improve, reflected in this conversation with the farm management team:

A12: “This is a very, very complex privately-owned company… I gave them a nickname, the ‘three steals farm’ (三偷牧场).”

MC: “‘Three steals’?”

A12: “Yes. Those with power, steal money. Those in charge of things, steal things. Those with no power, are lazy. (有权的人, 偷钱。中层干部, 偷东西。老百姓, 偷懒儿。) [...] This is a terrible farm. It is related to company culture…”

MC: “So you are managing, but you can’t manage the company’s culture”

A12: “We can’t change that! Company culture is a boss’ (CEO) culture [...] the biggest change we could make was changing the people, getting them to work more diligently [...].” He sighs. “We can only change some aspects of the people. But we can change the cattle’s health. Their nutrition is better, there is less lameness, mastitis. The cattle are full, healthy, and not dying. These are our changes. But [we, the farm management team] cannot change their company culture [...] This is why there are some things we cannot implement.”

In this dialogue, A12 conveys his belief that although some changes took place, company culture still limited the extent to which farm management is able to encourage workers to work more diligently and thereby positively impact animal care. This culture was further described by one member of the farm management team (A13) who stated that the ‘boss’ “does not really value raising cattle on this farm […] the farm only provides [a small portion] of the company’s milk supply.” Workers share material evidence such as the use of outdated equipment (e.g., broken, manual headlocks, broken hoof trimming chute) (A16; A17). MC observed how poor infrastructure occasionally led to rough handling, for example when undersized headlocks did not close properly on cattle with thicker necks.

A member of the farm management team (A12) commented on the lack of both integrity and transparency from the leaders, which conveyed a sense of not being valued by the company. This was also evident other three workers’ comments: “not getting paid on time” (A12), “underpaid and overworked” (A6), and too little time off work (A18; A6).

Worker A8 appeared frustrated by nepotism; for example, stating that the individual responsible for medication was the boss’ relative and so could not be held accountable for purchasing cheaper “fake” products, such as caustic paste which did not properly disbud calves. Additionally, the individual failed to purchase medication promptly, meaning veterinarian A5 felt the need to personally intervene: “This calf is about to die, so I bought this medicine. Using my own money! You know what I mean, to save her first.” Without clear direction and support from leadership, the responsibility of animal care on this farm seemed to depend, at least in part, upon personal ethics of the individual workers.

In addition to organizational culture issues mentioned above, workers also referred to other challenges (see Animal Care Context Section 3.1.) and described the infrastructure as ‘outdated’ (落后) and ‘archaic’ (原始). Despite these challenges, workers reported that there were improvements in animal care, attributing these to the competence of the new farm management team. Worker A18 shared that before the change in management there were daily cattle deaths, but “now we have better veterinarians so they can raise cattle well.” Members of the new management team came with relevant expertise, a change that was clearly respected by the workers; according to A9 this was a clear improvement as the previous farm manager “knew how to manage people but not cattle”.

#### 3.2.2. Organizational Culture: Farm B

The state-owned Company B (and thus also Farm B) placed emphasis on the CCP’s values and culture, reinforced by the mandatory presence of Party member workers who met frequently with other workers to disseminate Party teachings. As departmental leader and Party member B18 explained, the goal was “to spread positive energy from the Party”. For example, one meeting was about “iron man spirit/attitude” (铁人精神), a story of historical Chinese figures who used hard work, dedication, science, and a Chinese approach to ensure the growth of China’s oil industry; these attributes were then tied back to farm work, as illustrated by excerpt of B18′s speech below:

“Farm work can get monotonous and boring, but we need to have high spirits! To achieve ‘The Chinese Dream’ is not easy [...] We need to have an unyielding spirit. Be fearless of hard work/endure hardships (不怕苦)… and not forget about the hardworking spirit of the older generations…We need to become farm ‘iron man’, ‘cattle people’ (牛人).”

(‘cattle people’ is also slang for ‘awesome people’)

This Party culture appeared to improve worker motivation, sense of identity, pride, engagement, and spread the value of persevering through hardship and repetitive work, as well working towards a shared purpose of ‘The Chinese Dream’ (this is a concept and ideal of the great rejuvenation of the Chinese nation; it is promoted by Chinese Communist Party).

A sense of discipline and respect of hierarchy was evident among farm workers. Department leader B15 shared: “When the country’s political orders arrive, you need to execute without question”. This allowed for rapid implementation of top-down changes; for example, one of the team managers explained that when the government imposed stricter environmental regulations in 2015, the farm was quick to implement this directive by installing a manure recycling biogas facility (resulting in the usage of manure recycled stall bedding) and reducing usage of outdoor pens due to waste management concerns.

Farm B’s culture was also reflective of farm-level leadership. When asked what aspects of the farm he was most proud of, B5 (farm management team) smiled humbly and answered: “I think what we do best is identifying and solving problems… We need to ensure the cattle are most comfortable, the humans have good welfare, and our farm is prosperous […] If there is need for change, we need to do so immediately and be aware of our shortcomings.” Both younger and older workers shared a preference for younger workers, who were perceived to be more “aggressive/energetic” (闯劲儿), proactive in trying new ideas, motivated and productive compared to the older generation.

Workers shared how higher worker welfare translated to improved animal care. During discussions about worker entertainment activities (e.g., speech contests and singing festivities), B5 (farm management team) explained that “this is [farm B’s] specialty. Not just production related activities, but entertainment too. Give workers [higher] welfare, more free gifts, so everyone will see the farm as their home (以场为家). This actually means workers work more effectively.” Workers also shared how this aspect of farm culture attracted them to the farm and also improved animal care. Veterinarian B19 explained that he came to work at the farm because of

“high wages, good [worker] welfare […] Only after you ensure the people’s [quality of] life, can you ensure the cattle’s [quality of] life.” Worker B2 shared this sentiment: “they recently improved our meal plan, everyone is more motivated to work! [...] Cattle welfare is basically human welfare, only when humans live better can they take good care of the cattle.”

The conversations with, and the observations of, the farm workers showed evidence that both cattle comfort and humane treatment were viewed as important for productivity. Veterinarian departmental leader B11 shared “of course [there is hitting], but this occurs rarely…. The farm doesn’t allow hitting now. […] We rely on cattle for a living, how can we hit them?*”* Indeed, interactions observed between workers and cattle were calm, with few exceptions such as when cattle were forced to enter the hoof-trimming chutes, and for loading of adolescent bulls.

Leaders within each department shaped the departmental subculture. The reproduction department exemplified how a positive subculture may impact animal care outcomes. Many workers on the farm singled out this team’s unique culture and performance, including a member of farm management: “The percentage of infertile cows when I came was 12% [in 2016]. Manager B6 arrived in February 2017, after which we updated our SOPs. Now our infertility rate is 3%. Our reproductive team is doing really well, the best [out of all the farms at Company B]”.

The leader of the reproductive team (B7) explained that “The best management is militarized (军事化管理); I use this style in my management”. From participant observation, this involved a clear sense of shared purpose, frequent communication and reflection, discipline, and respect for the chain of command. Below is a field note excerpt taken during the team’s daily meeting:

The departmental leader stands in the corner of the cramped meeting room as the workers takes notes. He says: “we need to read lots and learn lots, not just about reproduction, but also history […] Don’t be short sighted. We must look at the bigger picture. Look at China’s dairy industry. We lack talent who can dedicate themselves. No matter which aspect [of cattle rearing], we need to take things to the next level, become world leaders […] Who are we depending on? Us, brothers. We must have these large ambitions and push through despite hardships.”

By tying daily work to the overall success of the Chinese dairy industry, workers were given a greater sense of purpose. These values and beliefs were not just communicated in meetings; MC observed workers (and especially the department leader) trying to reflect these values in their actions. B2 (from another department) shared how the reproduction team leader was unique in that he spent time individually with team members having “heart-to-heart chats”.

Another distinguishing feature of reproductive team’s culture was respect for chain of command, following directions given by the leader and SOPs. An example of the team’s militarized discipline was that workers would march to the dorms from the office in formation, which the department leader explained was to “set the tone”.

Trust and respect in the leadership was the norm in this department. When MC asked worker B9 about what aspects on the farm could be improved upon, he responded: “I’ve actually never thought about this… I just do my job properly […] The department leader is the one ‘taking the wheel’.” He went on to share how his role was to “make sure my execution skills are at 100%”.

### 3.3. Competency of Workers and Management

Competent individuals, in terms of formal education, personal experience, and work ethic, affected animal care. For example, farm management at Farm A complained that the company management’s lack of knowledge resulted in high morbidity and mortality. A13 (farm management) thought the cull rate set by the company management was unreasonably low. This meant as farm management, he could not cull enough cattle with low productivity, illnesses, and fertility issues, likely increasing on-farm deaths. He linked this to poor morale:

“[Workers] are frowning every day, of course their motivation is low. Additionally, medical fees are high, and many cattle are sustained by medication.” He further explained: “We provide training, and the workers agree (认可) with what they are taught. But they don’t execute it in practice. They know it is good to do this, but they say, ‘I can’t execute it, I have no energy’, there’s a vicious cycle.”

This illustrates how a lack of cattle knowledge at the company management level can ultimately lead to a vicious cycle of poor worker performance and animal care outcomes on the farm which is out of the farm management’s control.

Farm A’s management team appeared to make efforts in matching competent workers with positions appropriate to their skill set. To address high lameness rates, managers hired professional hoof trimmers to come and work for three weeks; MC observed that these experts handled cattle calmly and efficiently compared to farm workers who were not trained in this task. In another example, the farm manager commented on changing to a veterinarian with more calf experience—which according to workers improved calf health.

Farm management and technical staff (e.g., veterinarians, reproductive staff) on both farms commented on the importance of formal education for ensuring high standards of animal care. On Farm B, many departmental leaders were trade school graduates with diplomas in Animal Science. One such individual was department leader B17 who shared the benefits of hiring trade school graduates: “when it comes to hiring, I would definitely choose trade school [students]. […] firstly, they are obedient, secondly, they can do everything, thirdly they can endure hardships” (吃苦; literally ‘eat bitter’). However, workers also acknowledged that education needed to be coupled with experience. For instance, departmental leader B11 shared “Even if you have extremely high education […] you need to be able to apply this within farm context.” He argues that without “work experience”, “understanding farm procedures”, and “management experience”, “no one will listen to/respect you” (他不服你). Department leader B15 shared how the farm manager asked him to adapt the company SOPs for the farm because of his experience. This suggests that education needs to be coupled with relevant experience, especially for managers or technical staff.

On both farms there were indicators that employee education, experience and work ethic were respected. Department leader B15 shared how he studied each night, despite it being tiring, because “there is high competition, you need to know more than those you manage, you need to let them respect you!” (你得让他们服你啊!). His knowledge of cattle, animal science diploma, and constant learning earned him respect from his workers, helping him to manage even older workers. Workers on both farms shared that regardless of education (or “being cultured”; 有文化), eagerness to learn and a good work ethic (学习态度; 工作态度) played a major role in worker performance. For instance, a strong work ethic was observed in members of the new management team in Farm A; veterinarian A5 proudly shared: “I didn’t go to school. I trained myself as a ‘barefoot veterinarian’”; those who, despite being “uncultured/uneducated” (没文化), “use experience and skills to service the common folks” (‘barefoot doctor’, 赤脚医生, is a term that arose during the Cultural Revolution in reference to rural doctors, without medical training, who brought healthcare to places where urban doctors would not settle). He added as he patted a calf affectionately, “I am very dedicated to my technical skills. I will figure out every mistake I make. You need to know why you cured or failed to cure. That is the way of the successful.” When MC asked department leader A8 what makes a good worker, he shared “it’s not the education. It’s not the master/teacher. It’s your willingness to learn.”

One component of work ethic was being responsible. When asked about what makes him proud, department leader B15 said:

“As a veterinarian, I want to be competent (称职) at my job. I am not fooling around. […] Back when I worked as a veterinarian on [another farm at Company B], every year my performance was top 3 in the company! I feel like I am not letting down the cattle I am responsible for. No matter what job, if you want to master it, you need to devote experience and time to study it. You can’t ‘put away the cup after taking a tiny sip’ (浅尝辄止)”.

Similarly, a member of Farm A’s management team shares how the new farm manager’s high sense of responsibility combined with expertise bought improvements to animal care outcomes.

Notably, individual worker attitude and work ethics appeared to be influenced by organizational culture and norms. For example, on Farm B there appeared to be stronger norms regarding work ethic. Workers were generally more detail-oriented (e.g., picking up small pieces of rubbish from cattle feed even in the absence of supervisors), and interactions with cattle in the milking parlor were consistently gentle. Members of high performing departments (such as the reproduction department) were engaged (e.g., they appeared focused and took notes during group meetings), studious, and eager to learn. Within this department, one worker (B8) told MC they also wished they could do a research project to understand cattle reproduction, and another (B20) was reading about how to improve motivation in his spare time.

Workers on both farms reported challenges in finding competent and qualified workers. Departmental leaders on Farm B described challenges associated with the lack of talented individuals (especially younger workers) willing to dedicate themselves to farm work. This challenge was attributed by two departmental leaders B15 and B17 to be a consequence of China’s one child policy. This policy combined with the improvements in quality of life, was viewed to result in “only children” (独生子女) who were “given everything they wanted” (有求必应), “spoilt” (娇生惯养) and therefore “unable to adapt”, to do “dirty work” or “endure hardship” (吃苦) on farms. This gritty attitude and ability to endure hardship was repeatedly mentioned as critical to achieving success on both farms.

Qualified and educated workers might also be picky according to departmental leader B17, “people with college education might arrive and say: ‘the environment is not nice, I quit’”. For veterinarians this appeared to be a particularly relevant issue; team leader B2 (a veterinary school graduate) shared that most veterinary graduates prefer to work in city clinics where the pay was better. Departmental leader B15 also shared the difficulty to attract and retain veterinary students to the farm, even with a relatively high starting wage. Interestingly, on Farm B, all veterinary graduates were in leadership positions such as department leads, but only two of them were working on the veterinary team. The move to appoint educated workers directly into managerial positions (e.g., milking parlor department leader) seemed to be common; whereas, in the case of technical positions, they were required to work their way up to leadership positions (e.g., the reproductive departmental leader started off as a team worker). Veterinarians (兽医) was the title given to any staff who carried out medical care, regardless of qualifications. Veterinarians on both farms were mostly workers hired from nearby villages and trained on site. Departmental leader B15 felt that “there’s no requirements for becoming a vet in China”, arguing that this lack of qualified veterinarians resulted in preventable illnesses being left untreated, and that this hindered his ability to take a more proactive approach to disease prevention.

On Farm A, the farm management shared the view that qualified workers may not want to work on the farms due to low wages. Recognizing the difficulty in attracting and maintaining competent workers, Farm B’s management implemented a strategy of improving conditions via providing improved accommodation, food, wages, and worker benefits.

### 3.4. Incentives

Both farms used incentive systems that tied worker performance with rewards and punishments, providing a structure for encouraging certain outcomes. Workers reported that these incentives were effective in motivating behavior change. Both companies identified key performance indicators (e.g., increased profit; increased milk yield and quality) and utilized rewards to mutually benefit the farms and individual workers.

Farm managers on both farms reportedly increased performance appraisal (Farm A since the arrival of the new farm management team in March 2019, and Farm B since 2018). On Farm A, the farm management team’s compensation was based on farm profit, determined by milk quantity (kg) and quality (e.g., SCC). As such, the team saw the cattle’s well-being as instrumental to increased profit. Comments reflecting this mindset were conveyed by the farm management team. At a monthly meeting, one member of this team (A12) told workers, “see the cattle as your partner in earning money, when they are comfortable, they will produce more, and we will benefit” and another member (A13) added “if it is in the cow’s interest then it is correct.”

To achieve improved farm-level outcomes, Farm A’s new farm management team set merit-based pay for farm workers. The farm manager conveyed that since his arrival, he increased wages and merit-based pay for workers, including calf caregivers. Following wage increases, calf caretakers and veterinarians reported reductions in calf mortality and morbidity, and increased calf growth. One calf caregiver described: “I’m proud our calves grew more. The milk yield went up! […] When there’s more milk, workers earn more with merit-based income.” Similarly, Farm A’s reproduction team members shared how they took pride in their work and that when more cattle became pregnant, they earned more for their families.

Farm B had implemented a three-level performance appraisal system (三级考核) to align performance outcomes to company goals. In the first level, the company set farm-level key performance indicators (KPI) (e.g., daily yield should be 29.5 kg/cow/day; % cattle lying in stalls should be 80%) which determined how much each farm earns. In the second level, each department within Farm B was assessed based on relevant KPI (e.g., daily yield was a KPI for the milking parlor department; mastitis rates for the milking parlor department, veterinary department, environment department; and successful calving for maternity department). Within each department, the department leader was in charge of distributing wages to workers based on assessment of KPI. An example where having a performance outcome was viewed as important was conveyed by calf caretaker B14. In this case calf weight gain was tied to her monthly merit-based income, which was a strong motivator for this employee as she used her income to support her children. Below is a fieldnote excerpt as MC shadowed her:

“Before we fed the calves manually, using little buckets. Now we [use ad libitum feeding of acidified milk]. Each year is better than the last. The performance is better, the benefits are clear. You can see the effects of your work, and you can tell the worker’s ability. If [the calves are] fed well, [I’ll] earn better. With performance appraisal, if the calves gain weight, I will earn more. I quite like this job. Each month if the weight is above the goal, I am extremely happy!” She smiles broadly, looking very proud. “I’m so happy in my heart!” As we continue to feed the calf starter, she says to her calves “Eat more! Grow heavier!”

This kind of assessment was made possible given the data management department on Farm B, who collected data such as monthly calf weight gain, gait scores, and body condition scores for performance appraisals. These performance indicators were not measured on Farm A, in part, due to lack of a dedicated workers needed to generate this data.

Farm B also coupled training and education to performance appraisal, likely providing motivation for workers to implement what they learnt. For example, Farm B’s veterinarians were trained and tested on theoretical and technical knowledge and also clinical applications such as surgeries, while herders were trained and tested on low stress handling. Workers who received the top scores were given cash bonus as well as honorary red badges to wear (many recipients were seen proudly wearing them).

The effectiveness of the incentive system appeared to differ on the two farms, possibly due to differences in company culture, especially related to transparency, integrity, and fairness when it comes to finances. A member of farm management on Farm A complained “The key is the leader. His thinking determines the company’s direction. […] With performance indicators, it is important for the leader to clarify what happens if I achieve my goals, and what happens if I don’t. That’s it! Then keep your word. Don’t have internal conflicts. It’s that simple.” Workers shared how corruption within Company A meant even if the owner wanted to pay them the individual in charge of finances may not do so.

In contrast, on Farm B compensation appeared to be more transparent; all farm workers, including the farm managers, had their wages displayed on the canteen wall, and workers were observed comparing each other’s wages to see who benefited the most from the merit-based pay. This comparison appeared to be a strong motivator according to milker B20 who shared “you see each other’s wages, [the departmental leader tells you] why other people did so well, then I want to do even better”. However, not everyone saw this increase in performance review as ideal; department leader B15 shared how performance appraisals also increase rivalry and pit workers against each other. Departmental leader B17, who was in charge of paying his team members, emphasized the importance of transparency on Farm B: “If you are paying employees things must be completely clear and transparent. Everyone is here to earn money. Food comes first for the people, right? (民以食为天) […] So anything concerning money must be handled carefully.”

Punishments, such as fines, were used on both farms and were felt by some to be useful in discouraging poor behavior. For example, after the veterinary department leader on Farm B increased fines for veterinarians who made mistakes when writing their daily reports about cattle, these mistakes noticeably reduced. However, Farm A’s manager recognized that he could not enforce too many fines given that the base wage was low, and he expressed concern that implementing such an approach could result in some employees quitting their jobs.

Workers on Farm B (including departmental leaders and farm managers) were subjected to regular performance appraisals. B1, a member of farm management on Farm B indicated that they would be placing increasing emphasis on performance reviews.

“Everyone will start from the same starting point. [After performance appraisal a] team leader with good performance can be promoted to be department leader, while a department leader with poor performance will no longer hold that position anymore. Give them a sense of crisis and competition. Let them prove their technical skills and ability.”

Although the implementation of incentive systems appeared to help align worker performance with company goals, they were almost always profit focused, which can cause some tension with regards to animal care. For example, on Farm B it was viewed to be profitable to cull and sell the cattle alive vs. euthanizing. This may have resulted in some compromised cows suffering during transport and slaughter. On Farm B, any worker causing a cow to die was fined. Thus, workers stated that they chose to keep animals alive and cull them rather than euthanize.

## 4. Discussion

Our findings are not intended to be generalizable to all Chinese dairy farms, but the results may be transferable to farms with similar contexts. Chinese livestock farms are now viewed as being run more like a business compared to previous modes of traditional farming, meaning farm performance is closely tied to employee management and organizational culture [43]. Keeping in mind that farm performance and productivity do not necessarily indicate higher animal welfare, we discuss some animal welfare implications of our findings.

### 4.1. Organizational Culture

Leadership affects culture, which in turn impacts performance [44], employee attitudes [45], and job satisfaction [46]. While attitudes towards animals and work impact animal welfare [47], changing the attitudes of some workers is not sufficient in promoting a self-reinforcing culture of positive animal welfare [28]. Previous research has examined individual stakeholder attitudes using the theory of planned behavior [22,23], with the assumption that attitudes shape intentions, which in turn dictate behavior [48]. However, intention to change often does not result in actual behavior change [49]. Chinese society is highly collectivist and hierarchical [50], suggesting individual attitudes and intention may play less of a role in shaping behavior within a group context. It is thus critical to understand the farm context (e.g., organizational culture and norms) where behavior takes place.

Under top-down management systems, animal welfare initiatives may require approval and support of company and farm leaders [49]. For example, Farm B’s animal welfare related goals (such as reducing lameness and reducing SCC) were set by the company. These SOPs were adapted within each farm and department to ensure these were locally relevant and achievable. However, strong hierarchies coupled with Chinese cultural traits of deference to authority and conflict avoidance [51] may cause challenges. For example, subordinates may hesitate to provide critique or feedback to superiors, and workers may not have enough agency to make changes they find important.

Leadership approval is more likely if animal welfare improvement is viewed as beneficial (e.g., associated with increased productivity and profit; [52]). However, challenges can arise when outcomes focused on improving animal welfare conflict with economic outcomes, as was observed on Farm B where culling was viewed as financially beneficial compared to euthanasia.

Chinese culture places importance on interpersonal relationships (guanxi) [53], which facilitates corruption in certain contexts [54]. Such relationships in Company A appeared to hinder the farm management team’s ability to improve animal care.

### 4.2. Competence

Success of a dairy business depends on a competent workforce [55]. Competence was perceived to require a strong work ethic and knowledge of animal care (acquired through formal education, on-farm training, and personal experience).

As reported by workers, lack of skill can lead to issues such as poor detection of health problems. Classroom and online training can help increase Chinese livestock stakeholders’ knowledge of animal welfare issues [26]. Senior veterinarians, agriculture advisors, and scientific experts are considered influential by Chinese farmers [22]. For example, Farm A’s nutritionist was a well-respected advisor for farms across China. Although there is increasing Chinese literature about animal welfare [20], academic journals are not a common source of knowledge for Chinese livestock stakeholders [27]. Future efforts should focus on disseminating animal welfare knowledge from academia to influential leaders and workers—for example through extension and collaboration with veterinarians and nutritionists [30]. That said, it is important to realize that increased animal welfare knowledge does not necessarily lead to increased empathy [27] or changes in worker behavior [28], especially without appropriate incentives.

Competent workers were eager to learn and had a strong work ethic. High performance among Asian workers often includes drivers such as perseverance through hardship and a desire to become experts in the field (observed in Farm B’s reproductive team), and the acknowledgment that they can learn from past mistakes to transcend deficiencies (observed when veterinarian A5 perceived himself as uncultured but learning through mistakes) [56]. This type of ‘growth mindset’ (belief that ability can improve overtime) can be a predictor of higher performance compared to a ‘fixed mindset’ [57]. Grit (chiku, 吃苦, the ability to ‘eat bitter’ and persevere) is a key determinant of success [58] and considered by workers to be an important factor shaping farm success. These attitudes were articulated by workers on both farms and promoted in farm meetings, especially by leaders of high performing departments.

Though Chinese farmers did not rate ‘farmer well-being’ as highly important for improving animal welfare in surveys [22], qualitative research conducted in Finland [59] and Denmark [60] found that farmer well-being was critical to ensuring animal welfare. We found competent workers on these farms were attracted and motivated by improved working conditions, suggesting that, in similar contexts, improving worker welfare could improve animal welfare.

### 4.3. Incentive Systems

Effective incentive systems help increase work performance; linking performance appraisal to pay is common in Chinese businesses [61]. Increased pay appeared to motivate workers on both farms. That said, incentives tied to measures of profit will be only indirectly (and sometimes even negatively) associated with animal welfare; the animals are most likely to benefit if incentives are directly tied to welfare outcomes (e.g., Farm B linking successful calving to the maternity team’s wage). Sinclair et al. [24] recommended that animal welfare outcomes be included in key performance indicators. In farms with existing performance appraisal systems, animal welfare can be improved by incorporating animal-based welfare indicators (e.g., lameness and mastitis assessments were used in performance appraisal on Farm B).

Sinclair et al. [52] suggested that extrinsic motivators can shape behavior, even in the absence of intrinsic motivation. Creating clear and measurable goals during performance appraisal is associated with improved performance [62]. Our findings, similar to those of Armstrong [63], noted that accuracy, fairness, and transparency were important in performance appraisal. To improve worker satisfaction and performance, Peng and Tan [43] highlighted the need to include both material incentives (such as merit-based pay and gifts), and psychological incentives (such as having a pleasant work environment, and fun social activities).

There is currently no mention of ‘animal welfare’ in Chinese legislation [64,65], though farm animal welfare is affected by the Animal Husbandry Law (畜牧法; [66]) and slaughter regulations (e.g., in 2016 China introduced its first humane poultry slaughter regulation [67]). Within the dairy industry, leading processors such as Mengniu have created cattle welfare standards [68]. Relevant regulations and standards may be implemented by using such on-farm incentive systems.

### 4.4. Strengths and Limitations

How participants perceived MC shaped her findings. MC was often perceived as a curious “foreign student” (外国留学生) from the city. As such, most workers willingly explained farm life and animal care with her, but sometimes were uncomfortable sharing aspects of farm work which they perceived as “dirty” or embarrassing.

Ethnographic interviews and participant observations were valuable in engaging workers as they carried out daily practices, such as calf feeding, herding, and milking. Walking while interviewing stimulated richer discussion about the surroundings [69]. Additionally, more participants consented to informal, non-recorded conversations compared to formal, recorded interviews.

Immersive fieldwork allowed the researcher to participate in daily activities. While participants may have behaved differently in front of the researcher due to the observer effect (MC noted differences in behavior in certain participants on Farm A during the first two weeks of her visit), this was mitigated by spending more time with participants, developing trusting relationships, and becoming a normal presence. MC noted increased participant trust and willingness to share after receiving support from key decision-makers at each farm.

Triangulation using a variety of methods (e.g., interviews, observation, document collection, and participation) meant gaining a deeper and more holistic picture of the farms. Fieldwork involved interacting with a variety of workers. This included participants who might not have participated in surveys (e.g., hard to reach employees working directly with animals). These sampling issues are discussed by Li et al. [25]. Previous survey, interview, and focus group research on Chinese stakeholder’s perceptions of animal welfare may have suffered from self-selection of participants with an existing interest in the topic (e.g., Li et al. [25]). The farms and participants in the current study were not selected based on interest in animal care and welfare, reducing this selection bias.

The mini-ethnographic case study was practical, but results are limited by the relatively short time spent in the field. Due to limited time, we did not include company-level management; we acknowledge that these individuals can also impact animal care decisions. Given the annual and seasonal changes in dairy farm operations, MC’s one-month stay inevitably missed key events. The two farms differed in ownership (state-owned vs. privately-owned), herd size (1500 vs. 11,000), farm age, location, and many other factors; the findings from these farms are not generalizable to all Chinese farms, but key aspects may be transferable to farms with similar contexts. Ethnography has rarely been applied in the field of animal welfare [32], and our results illustrate how the application of this methodology can provide researchers with nuanced insights into the experiences of farm workers

## 5. Conclusions

Addressing complex animal welfare issues on farms requires an understanding of their cultural contexts. Qualitative approaches such as ethnography and participant observations can capture cultural factors that may be missed using other approaches. Our results indicated that farm worker satisfaction, competence, and motivation played a critical role in shaping animal care. We suggest that strategies to improve animal care include developing a positive organizational culture that unites individuals to pursue common goals, attracting and retaining competent workers (for example through improving worker welfare), and providing incentives for achieving animal care outcomes.

## Figures and Tables

**Table 1:** General farm information (accurate as of November 2019) for the two Chinese dairy farms used as study sites.

Farm Characteristic	Farm A	Farm B
Farm location	Central China	East China
Building date	2000s	2010s
Owned by	Privately-owned company	State-owned company
Number of workers	~50	~200
Worker age	30s–60s (average ~ 45)	20s–40s (average ~ 30)
Worker education	3 Trade school 10+ High school Rest: ≤ Middle school	2 Master’s 25 Trade school 30+ High school Rest: ≤ Middle school
Cattle breed	Holstein	Holstein
Herd size	~1500	~11,000
~milk yield/cow/d	25 kg	34 kg

## Data Availability

The data presented in this study are available on request from the corresponding author. The data are not publicly available in order to protect the confidentiality of the participants, and due to the personal and subjective nature of the data.

## Supplementary Materials

The following are available online at https://www.mdpi.com/article/10.3390/ani11051260/s1, Table S1: Initial interview guide, Supplementary material A: Longer quotes and excerpts in English and Chinese, Table S2: Final template, Supplementary material B: Sample of edited audit trail.
