# Supplementary material for: Employee Management and Animal Care: A Comparative Ethnography of Two Large-Scale Dairy Farms in China
Source: Animals (Basel). 2021 Apr 27;11(5):1260. doi: 10.3390/ani11051260 (PMC8147064; doi:10.3390/ani11051260)
Supplement: Supplementary file 1 [file animals-11-01260-s001.zip › animals-1081112-SI.pdf]

animals

MDPI

Table S1—Initial interview guide

This is the initial interview guide which was used during the 13 semi-structured interviews with farm workers on both farms.

Topic	Question	Translation
Demographics	你的老家是哪里的？	Where is your hometown?
	你在场上干了多久？	How long have you been working here?
	你为什么来这里/这个岗位工作？	Why are you working here/in this position?
Cattle well-being	对牛来说，什么是好的生活？	For cattle, what is a good life?
	什么对牛很重要？	What is important to cattle?
	牛喜欢/不喜欢什么？	What do cattle like/dislike
	你听过'动物福利'吗？	Have you heard of 'animal welfare'?
Cattle care on farm	场上有什么变化吗？	Have you seen any changes on this farm?
	有什么样的变化？	What kind of changes?
	你觉得场上哪方面做得比较好？	What do you think your farm is doing well?
	你觉得场上哪方面还可以提高？	What do you think your farm could improve on?
	哪方面让你最骄傲？	What are you most proud of in your work?

Supplementary material A —Longer quotes and excerpts in English and Chinese

Longer quotes and excerpts which appear in the results section are presented here, along with the original Chinese. The participant is represented by the anonymized identifier (e.g., A12).

Organizational culture: Farm A

A12: Organizational culture is the soul of an organization, something that unites people's hearts to do things.

A12: 企业文化是一个企业的灵魂，是凝聚人心，做事的。

Organizational culture: Farm A

A12: This is a very, very complex privately-owned company… I gave them a nickname, the 'three steals farm' (三偷牧场).

MC: 'Three steals'?

A12: Yes. Those with power, steal money. Those in charge of things, steal things. Those with no power, are lazy. (有权的人，偷钱。中层干部，偷东西。老百姓，偷懒儿。) [...] This is a terrible farm. It is related to company culture…

MC: So you are managing, but you can't manage the company's culture

A12: We can't change that! Company culture is a boss' (CEO) culture [...] the biggest change we could make was changing the people, getting them to work more diligently [...]." He sighs. "We can only change some aspects of the people. But we can change the cattle's health. Their nutrition is better, there is less lameness, mastitis. The cattle are full, healthy, and not dying. These are our changes. But [we, the farm management team] cannot change their company culture[...] This is why there are some things we cannot implement.

A12: 是个非常非常复杂的一个民有企业。。。我原来给他起了一个外号儿。叫三偷牧场。

MC: 三偷?

A12: 啊。有权的人，偷钱。中层干部，偷东西。(MC chuckles)老百姓，偷懒儿。[…]这是一个非常不好的牧场。和企业文化有关系…

MC: 就是你们管理，也管不了这个企业的人的文化。

A12: 改变不了！ 他企业文化是老板的文化[…]我们最大的能改变的就是这些人，现在去勤奋的劳动。[…]哎 ~ 改变人能改变一部分。但是能改变牛的健康。哦，这不是营养好了。瘸的少了。乳房炎少了。是吧。牛吃饱了，牛健康了，牛不死了。那就是我们改变的嘛。但是我们改变不了他们企业文化。[…]这就有一些，施展不开的。

Organizational culture: Farm B

B18: Farm work can get monotonous and boring, but we need to have high spirits! To achieve 'The Chinese Dream' is not easy [...] We need to have an unyielding spirit. Be fearless of hard work/endure hardships (不怕苦)….and not forget about the hardworking spirit of the older generations…We need to become farm 'iron man', 'cattle people' (牛人). (note on translation: 牛人, literally translated as 'cattle people', is also Chinese slang for "awesome people")

B18: 农场上的活也会单调，乏味。但是我们也应该有精神！要实现中国梦，不是那么容易。[…]我们要有不服输的精神。不怕苦。[…]不能忘了以前老一辈的人的吃苦精神。[…]我们也要做牧场'铁人'，'牛人'。

Organizational culture: Farm B

B5: I think what we do best is identifying and solving problems… We need to ensure the cattle are most comfortable, the humans have good welfare and our farm is prosperous […] If there is need for change, we need to do so immediately [and] be aware of our shortcomings.

B5: 我觉得我们做的最好就是发现问题。解决问题。[…]要做到让牛最舒服，人福利好，场好。要改立即改，认识到不足。

Organizational culture: Farm B

B5: this is [farm B's] specialty. Not just production related activities, but entertainment too. Give workers [higher] welfare, more free gifts, so everyone will see the farm as their home (以场为家). This actually means workers work more effectively.

B5: 对，这就是我们场的特色。不光是生产的活动，也有很多娱乐，生活。给员工福利，多给点免费的礼物，让大家以场为家。这样实际上人们工作起来也最有效。

Organizational culture: Farm B

B19: high wages, good [worker] welfare […] Only after you ensure the people's [quality of] life, can you ensure the cattle's [quality of] life.

B19: 工资高，福利好。[…]保障人的生活，才能保障牛的。

Organizational culture: Farm B

B2: they recently improved our meal plan, everyone is more motivated to work! [...] Cattle welfare is basically human welfare, only when humans live better can they can take good care of the cattle.

B2: 这边最近改善伙食，大家干劲儿大了！[…] 其实牛的福利就是人的福利，就是人过得好，才能管好牛。

Organizational culture: Farm B

B7: we need to read lots and learn lots, not just about reproduction, but also history […] Don't be short sighted. We must look at the bigger picture. Look at China's dairy industry. We lack talent who can dedicate themselves. No matter which aspect [of cattle rearing], we need to take things to the next level, become world leaders […] Who are we depending on? Us, brothers. We must have these large ambitions and push through despite hardships.

B7: 我们要多读书，多学习，不光是关于繁育，也一定要了解历史。[…]不要说把眼光放的很短。不要看的这么近。一定要看大格局。你看看中国的奶业。我们缺少大量的这个能钻研的人才。不管是哪一块儿[…]我们要把这些事情，推向高水平，推向世界前沿。[…]靠谁，就靠我们。兄弟们。就靠在坐的咱们。[…]要有这种大志向。一定要心存更高境界。然后你靠的什么？靠的是艰苦奋斗。

Competency of workers and management

A13: [Workers] are frowning everyday, of course their motivation is low. Additionally, medical fees are high and many cattle are sustained by medication." He further explained: "We provide training, and the workers agree (认可) with what they are taught. But they don't execute it in practice. They *know* it is good to do this, but they say, 'I can't execute it, I have no energy', there's a vicious cycle.

A13: [员工]每天愁眉苦脸，他精力是肯定降下来的。其次是药费，会很高，以药物维持的牛也特别多。。[…] 给他们培训了是吧，他们也认可了是吧，认可了，可是咱们现实当中没有去这么执行。[…] 他们**知道**这么做是好的。但是'我无法去执行。没有精力'。所以说等等一些方面，就是恶性循环。

Competency of workers and management

B15: As a veterinarian, I want to be competent (称职) at my job. I am not fooling around. […] Back when I worked as a veterinarian on [another farm at Company B], every year my performance was top 3 in the company! I feel like I am not letting down the cattle I am responsible for. No matter what job, if you want to master it, you need to devote experience and time to study it. You can't 'put away the cup after taking a tiny sip' (浅尝辄止).

B15: 我兽医技术，我要认为我是一位称职的技术兽医吧，就是我认为我不是一个什么糊弄工作的。[…]不管是我原来在[公司另一个牛场]从事兽医的时候，年年都是公司的，成绩都是前三名嘛.而且我觉得，我对得起我负责的牛. 不管是任何行业吧, 你想学精的话，还是需要花一些经历和时间去研究它。你不能浅尝辄止.

Competency of workers and management

A12: see the cattle as your partner in earning money, when they are comfortable, they will produce more and we will benefit"

A12: 你把牛看成合作或挣钱的伙伴，他们舒服了，就高产，有回报。

Setting up an effective incentive system

A12: The key is the leader. His thinking determines the company's direction. […] Just set goals. With performance indicators, it is important for the leader to clarify what happens if I achieve my goals, and what happens if I don't. That's it! Then keep your word. Don't have internal conflicts. It's that simple.

A12: 关键是一把手。他的思维，决定了企业的方向。[…]定目标就行了嘛。企业发展的目标。是吧。考核。你达到了我给你怎么着，你达不到了我给你怎么着，就行了。对不对。然后说话算数。但是中间不要内耗。就齐了！ 就这么简单。

Setting up an effective incentive system

B17: If you are paying employees things *must* be completely clear and transparent. Everyone is here to earn money. Food comes first for the people, right? (民以食为天) […] So *anything* concerning money must be handled carefully.

B17: 这个，给员工发工资的话*一定*一定要算得明明白白，清清楚楚的。别人出来上班都是挣的这份儿钱的。民以食为天嘛。[…]所以说对*任何*跟钱挂钩的事，一定要谨慎。

Setting up an effective incentive system

B14: "Before we fed the calves manually, using little buckets. Now we [use ad libitum feeding of acidified milk]. Each year is better than the last. The performance is better, the benefits are clear. You can see the effects of your work, and you can tell the worker's ability. If [the calves are] fed well, [I'll] earn better. With performance appraisal, if the calves gain weight, I will earn more. I quite like this job. Each month if the weight is above the goal, I am extremely happy!" She smiles broadly, looking very proud. "I'm so happy in

my heart!" As we continue to feed the calf starter, she says to her calves "Eat more! Grow heavier!"

B14: "以前是人工喂奶，用小盆儿。现在是[自由采食酸化奶]。一年比一年好。效益好，绩效好。就能看到干活儿的本质，体现出能力。喂的好，挣得好。绩效考核，体重增了，我的工资就增了。我挺喜欢这个工作。一个月增重超标，特别高兴!" 她骄傲地笑着说"心里老高兴！" 我们继续喂犊牛饲料时，她对牛说"多吃！喂重点儿！"

Setting up an effective incentive system

B1: Everyone will start from the same starting point. [After performance appraisal a] team leader with good performance can be promoted to be department leader, while a department leader with poor performance will no longer hold that position. Give them a sense of crisis and competition. Let them prove their technical skills and ability.

B1: 大家都在同一个起跑线上。[绩效考核后]组长干得好就能当上主管，主管当不好就不能当。给他们点危机感。让他们凭技术，凭能力。

Table S2 —Final template

This is the final template generated during data analysis.

Theme	Parent Code	Child Code
Organizational culture 企业文化 (OC)	OC-Leadership 领导 (LD)	LD-Integrity 诚信
		LD-Transparency 透明度
		LD-Nepotism 关系
		LD-Value worker welfare 员工福利
		LD-Value cattle 关注奶牛
	OC-Worker definitions 员工定义 (NV)	NV-Youthful 活力
		NV-Three steals 三偷牧场
	OC-Militarized management 军事化管理 (ML)	ML-Hierarchy
		ML-Shared purpose
		ML-Discipline 自律
		ML-Execution skills 执行力
		ML-Communication and reflection 沟通
Competent workers 称职员工 (CW)	CW-Education 教育 (ED)	ED-Trade school 专科
		ED-Uneducated 没文化
		ED-Training 培训
	CW-Experience 经验 (EX)	EX-Respect for knowledge 服气
		EX-Cattle knowledge 养牛
		EX-Management knowledge 员工管理
	CW-Attitudes/work ethics 工作态度 (AT)	AT-Shaped by organizational culture 企业文化
		AT-Eager to learn 学习态度
		AT-Grit 吃苦
		AT-Conscientious 认真
		AT-Responsible 责任心
	CW-Availability 缺少人才	
Incentive systems		

激励机制 (IS)	IS-Performance appraisal 绩效考核	
	IS-Consequence 后果 (CQ)	CQ-Rewards 奖励
		CQ-Punishments 处罚
	IS-Motivation 动力	
	IS-Accurate data 数据	
	IS-Accountability	
	IS-Implementation 执行	

Supplementary material B —Sample of edited audit trail

During data analysis, MC created an audit trail of commentaries on successive versions of templates. These reflections helped MC modify the template to better reflect what she was noticing in the data during analysis. This sample of the audit trail was modified to help improve ease and clarity of reading.

2020-07-22, 11:15 AM

Summary of major template changes:

I collapsed the previous 4 themes ('incentive systems'; 'execution'; 'organizational culture'; 'training and expertise') into 3 main themes ('incentive systems'; 'organizational culture'; 'competent workers')

Incentive systems as was quite distinct theme and remained relatively unchanged, while the other themes were modified as there were significant overlap of codes in 'execution', 'organizational culture', and 'training and expertise'.

Detailed notes:

The previous theme 'execution' covers when workers mention the importance of hierarchy, following rules, and having decision-making power, but as I am looking at the fieldnotes from when I was with the repro team at Farm B I feel these factors are also influenced by organizational culture and norms in a group. So I shifted the 'execution' codes under 'organizational culture'.

I was reading about one vet on Farm B saying he wants to be a 称职的兽医 ('competent veterinarian'). I feel worker 'competence' as a new theme can cover all the codes which once fell under 'training and expertise'. Moreover, by broadening the category from training and expertise to exploring overall worker competence, I can also include codes about work ethics and attitudes, which once fell under organizational culture.

I think talking about individual worker competence as a theme is helpful because in the organizational culture theme, I talk about how these norms shape individuals, but I still want to explore what makes an individual a 'competent worker' (e.g., Farm A's vet promoting ability to 吃苦/endure hardship).
